# Methodological comparisons for benefit-risk assessment in complex decision-making: applications and insights from a case study

**DOI:** 10.3389/fdsfr.2026.1792441

**Published:** 2026-06-10

**Authors:** Jian-Yu E, Kexin Zhu, Stéphanie Tcherny-Lessenot, Juhaeri Juhaeri

**Affiliations:** 1 Epidemiology and Benefit-Risk, Sanofi, Cambridge, MA, United States; 2 Epidemiology and Benefit-Risk, Sanofi, Morristown, NJ, United States; 3 Epidemiology and Benefit-Risk, Sanofi, Gentilly, France

**Keywords:** benefit-risk evaluation, case study, MCDA, qualitative analysis, quantitative analysis, SMAA

## Abstract

**Background:**

Benefit-risk assessment (BRA) can be challenging when integrating benefits, risks, and their relative importance as perceived by physicians or patients. This study aimed to compare eight BRA methodologies though the application and sights from a case study.

**Methods:**

Eight BRA methodologies were evaluated using data from European Public Assessment Reports, randomized controlled trials, and published literature on rimonabant, including two descriptive frameworks, two quantitative frameworks, and four metrics and estimation techniques.

**Results:**

Descriptive frameworks provided transparent, systematic approaches for structuring BRA and compared alternatives in a series of criteria assessment but lacked quantitative elements and stakeholder weighting. Quantitative frameworks extended these descriptive models by integrating objective data with subjective preferences, enabling the calculation of utility scores or acceptability indexes. Metrics enabled comparison of individual benefit and risk criteria, offering straightforward numerical evaluations.

**Conclusion:**

A combination of descriptive and quantitative frameworks allows for a comprehensive BRA, accommodating multiple criteria, stakeholder’s preferences, and uncertainties.

## Introduction

Benefit-risk assessment (BRA) is a fundamental component of the evaluation and decision-making, guiding the approval, labeling, and use of medicinal products. There are numerous BRA methodologies, which can be categorized into four main groups: descriptive frameworks, metrics, estimation techniques, and utility survey techniques (Mt-Isa et al., 2014). However, there is a lack of universal agreement and standard instructions on the most suitable methodology for structured BRA (Hughes et al., 2016). In particular, the practical application and implementation of these methodologies pose challenges, resulting in limited use within biopharmaceutical companies (FOOD AND DRUG ADMINISTRATION, 2023; Juhaeri, 2019; Kürzinger et al., 2020).

To demonstrate the practical application of BRA methods, we used rimonabant (Acomplia/Zimulti®) case study as an example. Rimonabant is a selective antagonist of cannabinoid type I (CB1) receptors indicated for weight loss in obese or overweight patients with co-morbidities (Van Gaal et al., 2005; Scheen et al., 2006). Approved in Europe in 2006, it was later withdrawn from the market in January 2009 on the grounds of a negative benefit-risk balance based on post-marketing data on the risks of serious psychological side effects, including suicide (European Medicines Agency, 2009). However, at the time of rimonabant’s withdrawal, a formal benefit-risk analysis using a quantitative method that integrates benefits, risks, and relative importance of benefits and risks according to physicians or patients had not been conducted. Note, this case study was performed to integrate real-life experiences to test and discuss BRA methodology, and it was not intended to replace or comment on any regulatory decisions for rimonabant.

This case study was part of a larger series of case studies commissioned by the Innovative Medicines Initiative’s Pharmacoepidemiological Research on Outcomes of Therapeutics by a European Consortium (IMI-PROTECT). The overarching objectives of IMI-PROTECT Benefit-Risk Group (IMI-PROTECT-BR) were to identify, characterize and test methods for use in BRA, including the data extraction and collation, modelling tools, and results presentation with the ultimate goal of creating a more systematic and structured approach to the benefit-risk evaluation to aid decision-making (Hughes et al., 2016; Juhaeri et al., 2011; Juhaeri et al., 2012). The BRA becomes complex when applied to decision-making contexts characterized by multidimensional features, including multiple benefit and risk criteria, the integration of different data sources, the incorporation of stakeholder preferences, and the need to consider trade-offs across competing criteria. This case study aimed to demonstrate the practical application of various methodologies for BRA using real-world data and to compare their strengths and limitations in a complex decision-making context. It also provided a pragmatic pathway for selecting and applying these methodologies, which was driven by decision context, data sources, the need to incorporate stakeholder preferences and uncertainty.

## Materials and methods

### Benefit-risk assessment methodologies

In this case study, eight BRA methodologies were tested, including two descriptive frameworks (Problem, Objective, Alternatives, Consequences, Trade-offs, Uncertainty, Risk and Linked decisions [PrOACT–URL] and Benefit Risk Action Team [BRAT]), two quantitative frameworks (Multi-Criteria Decision Analysis [MCDA] and Stochastic Multi-criteria Acceptability Analysis [SMAA]), four metrics and estimation techniques (Number Needed to Treat [NNT]/Number Needed to Harm [NNH], Impact Numbers [IN], and Benefit-risk Ratio [BRR], Probabilistic Simulation Method [PSM]).

All eight BRA methodologies were applied to the same rimonabant case study to enable a structured comparison. These methodologies were evaluated across multiple domains, including decision framework, use of meaningful and reliable data, incorporation of values and trade-offs, logical reasoning, and support for decision making. These domains were selected based on the established principles in BRA and their relevance to real-world applications (Tervonen et al., 2023; Pinto et al., 2023; Freyer et al., 2025).

### Data sources

The data for this case study were primarily obtained from publicly available sources, including the European Public Assessment Reports (EPAR) and published literature (European Medicines Agency, 2009). Results from six randomized controlled trials (RCTs) were used to supplement data collected from EPAR (Després et al., 2005; European Medicines Agency, 2006; European Medicines Agency, 2007; Van Gaal et al., 2005; Pi-Sunyer et al., 2006; Scheen et al., 2006; Topol et al., 2010; Rosenstock et al., 2008).

In addition to objective data from the RCTs, subjective data on relative importance of benefits and risks from different perspectives were gathered from team members using a direct elicitation method. Considering that the weighting of benefit and risk would vary across end-user groups, this exercise gathered input from a group of people comprising physicians, regulators and statisticians. For this exercise, physicians and regulators of the group provided medical and regulatory perspectives, while statisticians acted as the layman perspectives.

This direct elicitation method involved a questionnaire divided into medical/regulatory, and layman version (Supplementary Table S1). Participants were requested to score individual criterion between 0 (not important) and 10 (very important), with higher scores indicating greater importance. The scores from all participants were aggregated to calculate the average score, which was used to derive proportional weights. These relative importance weights were used in the MCDA and SMAA models. The Hiview 3 software (http://www.catalyze.co.uk/) was implemented for MCDA and the JSMAA software (http://smaa.fi/jsmaa) was implemented for SMAA.

## Results and discussion

These eight methodologies and justifications are introduced in Table 1 by their main categorizations. Overall, descriptive frameworks (PrOACT-URL and BRAT) focused on structuring and organizing evidence, quantitative frameworks (MCDA and SMAA) enabled integration of multiple criteria and stakeholder preferences, while metrics and estimation techniques (NNT, NNH, IN, BRR and PSM) provided the individual comparison of one benefit outcome to one risk outcome at a time.

**TABLE 1:** Overview of benefit-risk assessment methodologies and their justifications.

Framework	Methodology	Justifications
Descriptive	Problem, objective, alternatives, consequences, trade-offs, uncertainty, risk and linked decisions (PrOACT-URL)	- Structured, transparent and consistent in a BRA - Two competing, but very similar, methodologies - Simple, flexible, easy to understand - Efficient in establishing the decision-making context, gathering key information related to benefits and risks, deciding informed by experts judgment, and communicating the decision along with its rationale - Stakeholder preferences not included in either methodology - Not a decision analysis tool
	PhRMA benefit risk action team (BRAT)	
Quantitative	Multi-criteria decision analysis (MCDA)	- Both methodologies integrate objective treatment data with subjective preference data - Defining benefit-risk criteria through value trees and effects tables - MCDA provides a comprehensive approach to assessing benefit-risk balances, and is used to increase transparency for the decision process - SMAA is regarded as an extension to MCDA with the added simulation to handle data uncertainty - MCDA calculates an overall utility score, while SMAA provides an acceptability index that demonstrates its flexibility and robustness compared to MCDA
	Stochastic multi-criteria acceptability analysis (SMAA)	
Metrics and estimation technique	Number needed to treat and harm (NNT and NNH)	- Popularity and common use in the medical literature
	Impact numbers (IN)	- IN is conceptually similar to NNT but provides a public health perspective - Its applications have been promoted in literature and received considerable attention for simplicity
	Benefit-risk ratio (BRR)	- BRR is conceptually simple and general - The concept of taking the ratio of the magnitude of benefits to risks is tested
	Probabilistic simulation method (PSM)	- PSM allows for more complex benefit-risk models to be constructed taking into account various uncertainties in input values - PSM is flexible and has great potential when it comes to benefit-risk assessment

PhRMA, pharmaceutical research and manufacturers of america.

To illustrate their strengths and weaknesses, Table 2 summarizes the assessments of each methodology implemented in the case study by category in terms of 1) defining an appropriate frame, 2) using meaningful and reliable information, 3) availability of clear values and trade-offs, 4) logically correct reasoning, and 5) commitment to action. The comparisons of the eight methodologies in Table 2 highlighted the differences in how they structured the decision problem, integrated data, incorporated stakeholder preferences, and supported decision-making. These comparisons also highlighted where quantitative frameworks or metrics and estimation techniques can complement the descriptive frameworks.

**TABLE 2:** Assessment of benefit-risk assessment methodologies: practical considerations, strengths and limitations.

Methodology	Appropriate frame	Meaningful and reliable information	Availability of clear values and trade-offs	Logically correct reasoning	Commitment to action
PrOACT-URL	- Useful for addressing complex problems with multiple alternatives by breaking them into smaller, manageable criteria for objective and transparent decision-making - Introduces trade-offs by aligning criteria on the same scale, while guiding benefit-risk evaluations, uncertainty management, sensitivity analyses, and decision-maker risk attitude considerations - Comprehensive but can be time-intensive and shares common challenges with other benefit-risk assessment methodologies	Includes all relevant benefit-risk criteria with reliable clinical data	Explicit and transparent trade-offs are emphasized but demand exhaustive effort	Suitable for framing the problem rather than detailed analysis. Uncertainties in the data are addressed as part of the overall result	Encourages thoughtful consideration of related issues, ensuring transparency and clear audit trails. Ideal for regulatory decision-making, though exhaustive and time-consuming
BRAT	- Well-documented manuals and easy-to-use software - Involves stakeholders in selecting relevant outcomes	Focuses on presenting key outcomes based on stakeholder input	Allows indirect judgment of importance but struggles with presenting outcomes on different scales	Handles uncertainty via confidence intervals but may lead to arbitrary outcome selection	Provides structure and transparency but lacks flexibility for diverse data scales
MCDA	- Builds on PrOACT-URL, breaking complex problems into smaller criteria for efficient comparisons, with visual and numerical outputs - Flexible, allowing incorporation of new information as data becomes available - Limited by the need for precise criteria values and weights, often requiring detailed stakeholder discussions	Utilizes reliable data sources for benefit and risk criteria but may introduce bias in transforming data into utility scores and weighted values	Facilitates transparent evaluations of risk-benefit balances with results presented graphically and numerically	Assigns one value per criterion, limiting the handling of uncertainties inherent in medical data. Meta-analysis can mitigate this limitation	Allows decision-makers to structure problems objectively but is less suitable for medical decisions due to its limitations in addressing uncertainties
SMAA	- Based on PrOACT-URL but does not require precise stakeholder weightings - Accepts weight inputs as ranges or assumed equal during simulations - Free software (e.g., JSMAA) is available. Statistical knowledge enhances utility but is not mandatory	Utilizes reliable data sources, addressing uncertainties through ranges or distributions	Data are transformed into utility scores, and results are presented visually and numerically	Accommodates data in various forms (e.g., discrete values, ranges, distributions). Meta-analysis enables objective pooling of data between studies before using the results in this model	Simplifies decision-making by eliminating the need for precise weightings. Reliable and replicable but may struggle with large criteria sets
Impact number	Not designed to comprehensively frame problems but justifies evidence from clinical trials or databases	Only binary benefit-risk criteria are used. Best suited for safety-related criteria but less effective for complex framing	Impact numbers are presented on the same unit for benefit and risk but lack a trade-off method	Does not combine criteria or address uncertainties, except through recalculations	Useful for population-level decisions and resource allocation but less impactful for broader benefit-risk balancing
NNT	Not suitable for framing complex problems	Only binary criteria (e.g., efficacy or safety) are used, and clinical judgment is not required	Similar to impact numbers but struggles with rare events due to reliance on attributable risks	Limited to binary data with inherited uncertainties from attributable risks	Results lack population context and are difficult to communicate in multi-criteria analyses
BRR	Not suitable for framing complex problems	Limited to single benefit-risk comparisons, making it resource-intensive for broader analyses	Provides clear values and trade-offs but struggles with differing scales	Handles numeric data but does not address uncertainties	Effective for single comparisons but not for overall benefit-risk balancing in complex analyses
PSM	Not designed for problem framing but handles natural uncertainties effectively	Incorporates uncertainties using binomial distributions	Allows uncertainties to propagate through probabilistic simulations, providing an overview of parameter distributions	Handles numeric data and uncertainties well, even in complex evidence network of evidence where many other approaches would fail	Ensures proper handling of uncertain events and provides a comprehensive view of parameter distributions that may influence decisions

PrOACT-URL, Problem, Objective, Alternatives, Consequences, Trade-offs, Uncertainty, Risk and Linked decisions; BRAT, benefit risk action team; MCDA, Multi-Criteria Decision Analysis; SMAA, Stochastic Multi-criteria Acceptability Analysis; NNT, Number Needed to Treat; BRR, Benefit-risk Ratio; PSM, Probabilistic Simulation Method.

### Frameworks

#### Descriptive frameworks

PrOACT-URL was a generic decision-making guide with eight steps which required one to break down the issue in a systematic manner by defining the Problem, Objective, Alternatives, Consequences, Trade-offs, Uncertainties, Risk tolerance, and Linked decisions. Similarly, BRAT helped organize and summarize the evidence relevant to the benefit-risk decision through the following steps: define the decision context, identify benefit and risk outcomes and data sources, customize the framework, assess outcome importance, and display and interpret key benefit-risk metrics. As described in Table 2, these two descriptive frameworks were both found to provide a transparent, systematic framework for structuring a BRA by comparing alternatives in a series of criteria assessment. The result of descriptive frameworks was that benefit-risk outcomes could be summarized into a simplified value tree or effects table.

Coming up with the final value tree was an iterative process which often required stakeholders’ input on how to narrow down to the benefits and risks that have the most substantial impact as well as avoiding problems such as double-counting correlated endpoints. However, while the frameworks comprehensively outlined benefit and risk criteria and guided thought processes for discussing key points and communicating issues, they remained descriptive and lacked a formal quantitative analysis for benefit-risk evaluations. Stakeholder’s preferences on criteria were not required in either descriptive frameworks and neither PrOACT-URL nor BRAT was a decision analysis tool.

#### Quantitative frameworks

MCDA and SMAA were decision analysis tools that extended the information gathered in the descriptive frameworks by providing a quantitative way to integrate objective treatment effects data with subjective preference data (e.g., relative importance). Similar to the qualitative frameworks, the context of the problem was defined, and value trees and effects tables were constructed to define the main benefit-risk criteria to enter into the model which generated an overall utility score. Sensitivity analysis could be performed, allowing for different criteria to be assigned a range of weights (Table 2).

For the MCDA, the comparison for this case study was rimonabant vs. placebo. Benefit-risk data were obtained, and value trees and effects tables were constructed in a similar method to the PROACT-URL framework. Since the preference weights were derived from a basic website survey among team members (direct elicitation of medical/regulatory or layman preferences), the results were sensitive to stakeholder weights, which should only be viewed as an example of how this technique could be implemented. Specifically, sensitivity tests indicated that small adjustment in the weights assigned to benefits or risks would alter the preferred treatment. Note, although several value trees were created, a value tree using summarized data from RCTs analyzed by meta-analysis was described. This method was beneficial as it could account for the uncertainties and population differences across the trials.

The MCDA results were also sensitive to the type of weighting used (e.g., regulatory vs. layman). Using the value tree based on meta-analysis data with layman weighting, the total weighted score favored rimonabant. However, unlike the regulatory weighted model, sensitivity tests of the layman model showed that it was relatively stable with respect to moderate changes in weightings for benefits and risks.

Compared to MCDA, SMAA was better equipped to handle uncertainty within the data by using simulations (Table 2). SMAA was also more flexible, as it did not require explicit preference information from stakeholders upfront; instead, the model could assume a uniform weight distribution across criteria. If ranking preferences were available, the model could incorporate these rankings as well.

Unlike MCDA’s utility score, SMAA provided an acceptability index for a particular ranking. Data were transformed into utility score using linear criteria function. Instead of using the fixed scale defined by stakeholders in MCDA, SMAA method generated the fixed scales using the 95% confidence intervals. This method produced a common scale to allow comparison between risks and benefits. Final results were easily interpretable in both graphical and numerical form using free and easy to use software (i.e., JSMAA, http://www.smaa.fi/).

#### Metrics and estimation techniques

Metrics were used to numerically express the value of treatment effects. The IN described the number of people affected by a binary event within a population and could include measures to estimate the effect of interventions over a given period (e.g., the number of events prevented in a population [NEPP] or the population impact number of eliminating a risk factor [PIN-ER-t]). For example, assuming rimonabant was implemented in the United Kingdom obese population, its potential impact could be extrapolated based on clinical trial results. However, in the case of rimonabant, these metrics were not particularly useful for benefit-risk decision-making, as they might not accurately reflect real-life conditions where multiple interventions could exist and where multiple outcomes needed to be considered. Additionally, it was challenging to interpret and integrate multiple criteria and incorporate uncertainties.

The NNT/NNH were also known as exposure impact numbers. Ideal treatments had a very low NNT on benefit criteria and a very high NNH on risk criteria. These metrics were relatively straightforward to calculate for each benefit-risk criterion; however, similar to NEPP or PIN-ER-t, they lacked direct relevance for benefit-risk decision-making and, given the numerous criteria involved, were difficult to communicate effectively. The BRR was often useful for directly comparing a benefit to a risk and could be weighted for relative importance (Table 1). However, this metric could not incorporate uncertainties, might have scaling issues, and required a separate BRR calculation for each benefit-risk criterion. Consequently, it could be too restrictive when multiple criteria were involved. One technique that could address the limitation of metrics in incorporating uncertainties was the PSM (Table 1). PSM method repeatedly samples each parameter of interest to capture natural variation, allowing for calculation of 95% confidence intervals. However, even with uncertainty incorporated, these metrics remained best suited for specific public health questions (e.g., resource allocation) and might not be ideal for complex BRA in regulatory settings, such as the case of rimonabant, which presented a controversial therapeutic promise alongside safety concerns.

These study findings are broadly aligned with recent literature showing that no single BRA methodology is suitable for all decision settings, and that the appropriate method selection depends on the decision context, available data sources, and the need to incorporate preference and uncertainty. Tervonen et al. reported the expert consensus in good practice for developing quantitative BRA, emphasizing the importance of the research question, model development, preference elicitation, data analysis and communication of results (Tervonen et al., 2023). Similarly, Sullivan et al. introduced an industry operating model for structured BRA, which outlined a tripartite classification of descriptive, semi-quantitative, and quantitative BRA approaches (Sullivan et al., 2023). They also highlighted the value of using a systematic framework across the drug development stages, e.g., from first-time-in-human (FTiH) studies to regulatory submission, while also noting practical challenges in routine implementation (Sullivan et al., 2023). More recently, a review by Suzumura et al. further showed that the BRA approaches remained heterogeneous in scope, methods and reporting, identifying 129 approaches for conducting BRA and 37 visual tools for reporting BRA results, which limited the consistency in real-world application (Suzumura et al., 2024). Against this backdrop, the present study added value by applying the eight BRA methodologies to the same case study and enabling direct comparison of descriptive, quantitative frameworks and metrics and estimation techniques under a complex decision context. By applying multiple BRA methodologies to a single case study, this study provided practical insights into their comparative performance and highlighted key considerations for their implementation in real-world settings, and therefore helping to bridge the gap between methodological development and practical implementation.

## Limitations

This case study was not intended to evaluate the past regulatory decisions regarding rimonabant. The choice of rimonabant, a drug withdrawn due to psychiatric adverse events (e.g., depression and anxiety), reflected the need for a well-documented example with a complex benefit–risk profile; however, it might not capture the complexity and diversity of decision-making for other anti-obesity medications. While this example provided useful context for comparing BRA approaches, we acknowledged the ethical sensitivity associated with such a case and noted that the study focused on methodological considerations in nature.

In addition, the trade-offs and preferences were collected directly from team member surveys rather than through formal methods. Additionally, the case study was limited to the eight main methodologies in BRA. The study does not advocate any specific methodology but aims to share insights on the strength and weakness of the methodologies tested in a real-world case.

## Conclusion

This case study provides a valuable real-world example of implementing eight methodologies for BRA in the context of complex decision making. By applying these methodologies to the same case study, the analysis enables a direct comparison of their roles, strengths, and limitations under a multifaceted decision context. This study finds that no single benefit-risk methodology can fully capture all aspects of the assessment; rather, each approach contributes different strengths in structuring the problem, integrating data, incorporating stakeholder preferences, and addressing uncertainty. The choice of a single approach or a combination of approaches should be guided by the complexity of the decision problem. In this case study, a combination of a descriptive framework supplemented by quantitative models allows the incorporation of multiple benefit and risk criteria, reflects varying stakeholder preferences, and addresses uncertainty, leading to a comprehensive BRA. Overall, our study highlights the value of a pragmatic and context-driven approach to BRA and provides practical insights into selecting and applying methodologies in real-world settings.

## Data Availability

The original contributions presented in the study are included in the article/Supplementary Material, further inquiries can be directed to the corresponding author.
