# Supplementary material for: Methodological comparisons for benefit-risk assessment in complex decision-making: applications and insights from a case study
Source: Front Drug Saf Regul. 2026 Jun 10;6:1792441. doi: 10.3389/fdsfr.2026.1792441 (PMC13290793; doi:10.3389/fdsfr.2026.1792441)
Supplement: Supplementary file 1 [file Table1.docx]

Supplementary Table 1. Questionnaire for utility elicitation

|  | **0 (Not important)** | **1** | **2** | **3** | **4** | **5** | **6** | **7** | **8** | **9** | **10 (Very important)** |
| --- | --- | --- | --- | --- | --- | --- | --- | --- | --- | --- | --- |
| **1. In case of weight losing medication, how would you rate the importance benefit and risk?** | | | | | | | | | | | |
| Benefit |  |  |  |  |  |  |  |  |  |  |  |
| Risk |  |  |  |  |  |  |  |  |  |  |  |
| **2. In the use of rimonabant, a drug designed for weight lost, how important are the following benefits?** | | | | | | | | | | | |
| Success in losing and maintained 10% lost of bodyweight |  |  |  |  |  |  |  |  |  |  |  |
| Improvement in cholesterol control |  |  |  |  |  |  |  |  |  |  |  |
| Reduce triglyceride levels |  |  |  |  |  |  |  |  |  |  |  |
| Reducing waist circumference |  |  |  |  |  |  |  |  |  |  |  |
| Improvement in diabetes control |  |  |  |  |  |  |  |  |  |  |  |
| Lowering blood pressure |  |  |  |  |  |  |  |  |  |  |  |
| Reducing incidence of metabolic syndrome |  |  |  |  |  |  |  |  |  |  |  |
| **3. With regards to cholesterol control, how important is the following markers of cholesterol?** | | | | | | | | | | | |
| Total Cholesterol (Sum of Cholesterol level) |  |  |  |  |  |  |  |  |  |  |  |
| HDL cholesterol ("Good Cholesterol") |  |  |  |  |  |  |  |  |  |  |  |
| LDL cholesterol ("Bad Cholesterol") |  |  |  |  |  |  |  |  |  |  |  |
| HDL/LDL cholesterol ratio |  |  |  |  |  |  |  |  |  |  |  |
| **4. With regards to measurements of diabetes control, how would you rate the importance of the following markers?** | | | | | | | | | | | |
| Fasting Glucose (Use in diagnosis of diabetes) |  |  |  |  |  |  |  |  |  |  |  |
| Fasting Insulin (Measurement of Insulin production) |  |  |  |  |  |  |  |  |  |  |  |
| Insulin resistance |  |  |  |  |  |  |  |  |  |  |  |
| Changes in HbA1C (Overall Diabetes control over 120 days) |  |  |  |  |  |  |  |  |  |  |  |
| **5. With regards to blood pressure control. How would you rate the importance of the following?** | | | | | | | | | | | |
| Systolic blood pressure (Top measurement) |  |  |  |  |  |  |  |  |  |  |  |
| Diastolic blood pressure (Bottom measurement) |  |  |  |  |  |  |  |  |  |  |  |
| **6. With regards to weight losing medication, rimonabant, independent to its benefit. How would you rate the importance of avoiding potential negative effect in following body system?** | | | | | | | | | | | |
| Infection and Infestation |  |  |  |  |  |  |  |  |  |  |  |
| Psychiatric disorder |  |  |  |  |  |  |  |  |  |  |  |
| Nervous system disorder, for example dizziness or neuralgia |  |  |  |  |  |  |  |  |  |  |  |
| Vascular disorder, e.g. hot flushes |  |  |  |  |  |  |  |  |  |  |  |
| Skin and subcutaneous tissue disorder |  |  |  |  |  |  |  |  |  |  |  |
| Musculoskeletal disorder |  |  |  |  |  |  |  |  |  |  |  |
| Injury, poising or procedure related complication |  |  |  |  |  |  |  |  |  |  |  |
| Severe adverse events (i.e. events that caused irreversible damage or require hospitalization) |  |  |  |  |  |  |  |  |  |  |  |
| **7. How would you rate the importance of the following reported side effects associated with rimonabant?** | | | | | | | | | | | |
| Upper respiratory tract infection |  |  |  |  |  |  |  |  |  |  |  |
| Gastroenteritis viral |  |  |  |  |  |  |  |  |  |  |  |
| Anxiety |  |  |  |  |  |  |  |  |  |  |  |
| Insomnia |  |  |  |  |  |  |  |  |  |  |  |
| Mood alternation with depressive symptoms |  |  |  |  |  |  |  |  |  |  |  |
| Depressive disorders |  |  |  |  |  |  |  |  |  |  |  |
| Irritability |  |  |  |  |  |  |  |  |  |  |  |
| Parasomnia |  |  |  |  |  |  |  |  |  |  |  |
| Nervousness |  |  |  |  |  |  |  |  |  |  |  |
| Sleep disorders |  |  |  |  |  |  |  |  |  |  |  |
| Dizziness |  |  |  |  |  |  |  |  |  |  |  |
| Memory loss |  |  |  |  |  |  |  |  |  |  |  |
| Hypoesthesia |  |  |  |  |  |  |  |  |  |  |  |
| Sciatica |  |  |  |  |  |  |  |  |  |  |  |
| Hot flushes |  |  |  |  |  |  |  |  |  |  |  |
| Nausea |  |  |  |  |  |  |  |  |  |  |  |
| Diarrhoea |  |  |  |  |  |  |  |  |  |  |  |
| Vomiting |  |  |  |  |  |  |  |  |  |  |  |
| Pruritus |  |  |  |  |  |  |  |  |  |  |  |
| Hyperhydrosis |  |  |  |  |  |  |  |  |  |  |  |
| Tendonitis |  |  |  |  |  |  |  |  |  |  |  |
| Muscle cramp |  |  |  |  |  |  |  |  |  |  |  |
| Muscle spasms |  |  |  |  |  |  |  |  |  |  |  |
| Influenza |  |  |  |  |  |  |  |  |  |  |  |
| Asthenia/Fatigue |  |  |  |  |  |  |  |  |  |  |  |
| Joint sprain |  |  |  |  |  |  |  |  |  |  |  |
| Contusion |  |  |  |  |  |  |  |  |  |  |  |
| Fall |  |  |  |  |  |  |  |  |  |  |  |
| Death |  |  |  |  |  |  |  |  |  |  |  |
| Overall Psychiatric disorder |  |  |  |  |  |  |  |  |  |  |  |
| Cardiac disorder |  |  |  |  |  |  |  |  |  |  |  |
| Urinary disorder |  |  |  |  |  |  |  |  |  |  |  |
| Road traffic accident |  |  |  |  |  |  |  |  |  |  |  |
| Overall severe psychiatric disorder (e.g. depression require hospital admission) |  |  |  |  |  |  |  |  |  |  |  |
| Heart disorder (e.g. Heart attack) |  |  |  |  |  |  |  |  |  |  |  |
| Disorder of kidney and bladder |  |  |  |  |  |  |  |  |  |  |  |
| Road traffic accident |  |  |  |  |  |  |  |  |  |  |  |
